# Appraising clinical applicability of studies: mapping and synthesis of current frameworks, and proposal of the FrACAS framework and VICORT checklist

**DOI:** 10.1186/s12874-021-01445-0

**Published:** 2021-11-14

**Authors:** Quoc Dinh Nguyen, Erica M. Moodie, Philippe Desmarais, Robert Goulden, Marie-France Forget, Eric Peters, Sahar Saeed, Mark R. Keezer, Christina Wolfson

**Affiliations:** 1 Division of Geriatrics, Centre hospitalier de l’Université de Montréal, 1000, Saint-Denis, Montreal, Quebec H2X0C1 Canada; 2 Centre de recherche du Centre hospitalier de l’Université de Montréal, Montreal, Canada; 3 Department of Epidemiology, Biostatistics, and Occupational Health, McGill University, Montreal, Canada; 4 Department of Medicine, McGill University, Montreal, Canada; 5 Department of Anesthesia, Centre hospitalier universitaire Sainte-Justine, Montreal, Canada; 6 Department of Infectious Disease, Washington University in St. Louis, St. Louis, USA; 7 Departments of Neurosciences & Social and Preventative Medicine, Université de Montréal, Montreal, Canada; 8 Neuroepidemiology Research Unit, Research Institute of the McGill University Health Centre, Montreal, Canada

**Keywords:** Quality assessment, External validity, Generalizability, Impact, Evidence-based practice

## Abstract

**Background:**

Not all research findings are translated to clinical practice. Reasons for lack of applicability are varied, and multiple frameworks and criteria exist to appraise the general applicability of epidemiological and clinical research. In this two-part study, we identify, map, and synthesize frameworks and criteria; we develop a framework to assist clinicians to appraise applicability specifically from a clinical perspective.

**Methods:**

We conducted a literature search in PubMed and Embase to identify frameworks appraising applicability of study results. Conceptual thematic analysis was used to synthesize frameworks and criteria. We carried out a framework development process integrating contemporary debates in epidemiology, findings from the literature search and synthesis, iterative pilot-testing, and brainstorming and consensus discussions to propose a concise framework to appraise clinical applicability.

**Results:**

Of the 4622 references retrieved, we identified 26 unique frameworks featuring 21 criteria. Frameworks and criteria varied by scope and level of aggregation of the evidence appraised, target user, and specific area of applicability (internal validity, clinical applicability, external validity, and system applicability). Our proposed Framework Appraising the Clinical Applicability of Studies (FrACAS) classifies studies in three domains (research, practice informing, and practice changing) by examining six criteria sequentially: Validity, Indication-informativeness, Clinical relevance, Originality, Risk-benefit comprehensiveness, and Transposability (VICORT checklist).

**Conclusions:**

Existing frameworks to applicability vary by scope, target user, and area of applicability. We introduce FrACAS to specifically assess applicability from a clinical perspective. Our framework can be used as a tool for the design, appraisal, and interpretation of epidemiological and clinical studies.

**Supplementary Information:**

The online version contains supplementary material available at 10.1186/s12874-021-01445-0.

## Introduction

Not all health research findings are translated into clinical or public health interventions [1]. Many reasons for lack of implementation relate to research quality and validity [2–5]. Excellent frameworks have been developed to assess the quality of epidemiological and clinical research by predominantly assessing the internal validity of research findings (e.g., confounding, selection and measurement biases) [6–9]. What determines high quality and validity research may not, however, directly determine what is most impactful [10]. The appraisal of applicability, whether study results can impact practice, demands an expanded set of considerations. The cumulative nature of evidence and of the strength of evidence is the focus of many important frameworks, most notably GRADE (Grading of Recommendations, Assessment, Development and Evaluations) [11] used to synthesize evidence and formulate clinical recommendations [12]. The appropriateness and relevance to clinical practice of research questions or findings may need to be considered; not all exposures, interventions, associations, and outcomes are equally informative to practice [13, 14]. External validity is another critical focus when applying study results to specific practice and population contexts (generalizability and transportability) [15–18].. Implementation science and economic considerations also factor in the practical application of research [19–22].

Although current frameworks cumulatively cover many important facets of applicability, the specific criteria to assess applicability may vary by the type of research and evidence, and by the stakeholders involved: researchers, clinicians, decision-makers and policymakers. Clinical applicability can be defined as the potential of study findings to inform or directly alter current clinical practice at the individual level. Due to their wide scope, it is unclear whether existing frameworks can concisely assist clinicians in differentiating between studies that change practice, inform practice, or are not clinically applicable. As clinicians must evaluate an ever-expanding research output, there is a need to better identify criteria that may be used to gauge applicability, in particular clinical applicability.

In this two-part study, we conducted a broad literature review to identify, map, and synthesize existing frameworks and criteria pertaining to the applicability of studies. Drawing from this review, current concepts and debates in epidemiology [23–26] and clinical research [13, 27], and iterative discussions and testing, we developed a concise tool to classify and improve the applicability of studies, with an emphasis on the clinical perspective. FrACAS, our proposed Framework to Appraise the Clinical Applicability of Studies and its checklist (VICORT) are introduced and discussed.

## Methods

### Search, thematic mapping, and synthesis of available frameworks

We searched PubMed and EMBASE (Ovid) databases since their inception for articles reporting on frameworks appraising the general “applicability” of research findings on November 12, 2020. The eligibility criteria were articles (i) featuring a unique tool, instrument, checklist, or framework (ii) focused on the applicability to practice of (iii) health research evidence, and (iv) published in English. We excluded articles that solely featured a review of frameworks, the application of an existing framework, or were restricted to a specific condition or discipline. Due to the potential multiple understandings of “applicability,” we used combinations of keywords in titles and abstracts to maximize the comprehensiveness of article selection as previously done by others on the topic of applicability [15, 16]; the full search strategy is detailed in the Additional file 1: Methods. Duplicates were removed, titles and abstracts were screened independently by two authors (PD and QDN). We supplemented remaining articles with references in reviews and retrieved articles. Articles were assessed in full to identify unique frameworks. PD and QDN performed conceptual thematic analysis [28] using preliminary themes that were refined iteratively to map the frameworks and to synthesize criteria of applicability by stakeholders. Disagreements were resolved by consensus.

### Development of framework for clinical applicability

**Fig. 1:**
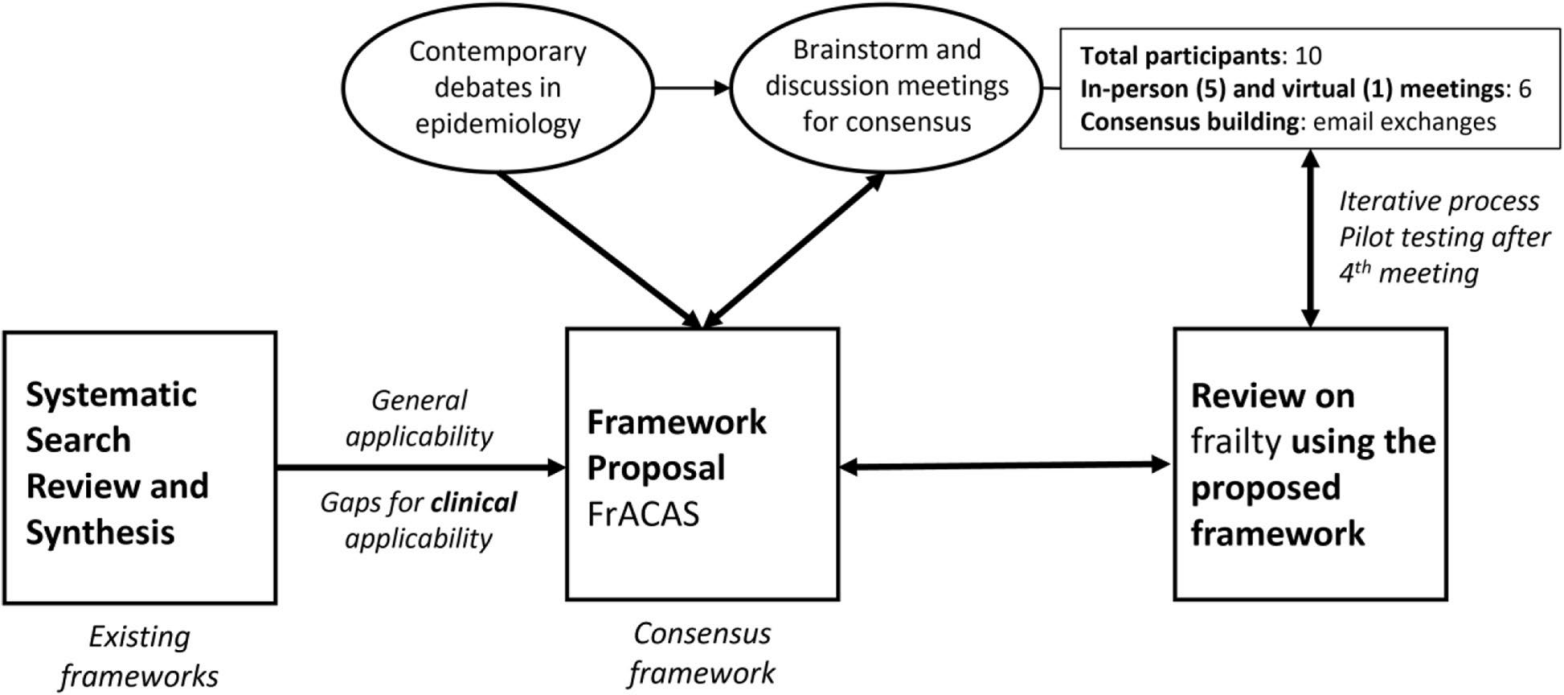
Process and inputs for the development of the Framework for Appraising the Clinical Applicability of Studies (FrACAS)

As illustrated in Fig. 1, we developed our framework by integrating four major inputs: contemporary debates in epidemiology and clinical research, brainstorming and discussion meetings, comparison with existing frameworks for appraisal of clinical applicability, and pilot application testing of our framework. Ten clinicians, researchers, and methodologists with expertise in multiple substantive domains of clinical practice and research (intensive care, pediatrics, internal, emergency, and geriatric medicine), as well as epidemiology, biostatistics, qualitative, and translational research, participated in a total six brainstorming and discussion meetings (in-person and virtual). Each meeting introduced a preliminary version of the framework which was discussed and progressively altered between each subsequent meeting. After the fourth meeting, pilot testing of the preliminary framework was conducted in a mapping review on the clinical applicability of frailty on 10 articles (forthcoming), and feedback was incorporated to the following iteration. Not all participants attended all meetings, and although formal Delphi methodology was not employed, versions of the framework were iteratively refined and circulated by email to reach the final consensus framework.

## Results

### Analysis, mapping, and synthesis of frameworks for applicability

We identified 4622 references, of which 1324 were duplicates and 3265 were excluded following the screening of titles and abstracts, leaving 33 for assessment. Thirty additional references were identified in reviews and references from retrieved articles; we assessed 63 full text articles and included 26 unique frameworks. Additional file 1: Fig. A1 presents the flowchart for article selection.

#### Description and analysis of frameworks

Table 1 presents the 26 frameworks and their predominant focus [6, 7, 11, 17, 18, 22, 29–55]. Frameworks were published between 1999 and 2021 in epidemiological, clinical, public health, policy, and decision-making journals. Although we only included frameworks related to applicability, the focus varied widely from the quality of clinical practice guidelines (CPG, AGREE I-II [29, 30]), quality and strength of recommendations (GRADE) [11], use of evidence to inform health decisions (GRADE EtD) [18], applicability of prediction model studies (PROBAST) [43], applicability of randomized trials (PRECIS) [41] and health technology assessments (HTA) [47, 55]. Due to distinct purpose and focus in appraising applicability, the complexity of frameworks and the number, nature, and level of criteria detail within frameworks also varied. Some frameworks featured a simple list of key criteria [50, 53] whereas others elaborated on a full system of domains, criteria, and appraisal processes (e.g., RE-AIM [22, 44], GRADE [11], PRECIS [41], RoB2 [7], RoBINS-I [56], Atkins et al. [48]); some adapted to specific concepts and disciplines (GRADE EtD) [18, 34–38]. After comparative analysis of frameworks, we identified three dimensions explaining the variability which we used to map the frameworks and criteria:

- The primary intended target user or stakeholders (researchers, clinicians, and decision-makers);
- The evidence type appraised and its level of aggregation, from fundamental research to CPG;
- The areas of applicability: internal validity, clinical applicability for individual patients, external validity, and applicability at the system level.

**Table 1:** Frameworks for appraising applicability of studies

Framework and/or First Author		Journal	Framework Focus	Year of Publication
AGREE I	Cluzeau et al. [29]	Quality and Safety in Health Care	Quality of CPG	2003
AGREE II	Brouwers et al. [30]	Canadian Medical Association J		2010
AGREE-REX	Brouwers et al. [31]	JAMA Network Open	Quality of CPG recommendations	2020
ASTAIRE	Cambon et al. [32]	BMC Public Health	Transferability of health promotion interventions	2013
EVAT	Khorsan et al. [33]	Evidence-Based Complementary and Alternative Medicine	Evidence for clinical decision-making	2014
GRADE	Guyatt et al. [11]	British Medical Journal	Quality of evidence and strength of recommendations	2008
GRADE EtD				
Clinical recommendations	Alonso-Coello et al. [18, 34]	British Medical Journal	Evidence usage in a structured and transparent way to inform and adapt clinical and public health decisions	2016 2016
Coverage decisions	Parmelli et al. [35]	Int J of Tech Assessm in Health Care		2017
Diagnostic/screening tests	Schünemann et al. [36]	Journal of Clinical Epidemiology		2017
Health system and public health	Moberg et al. [37]	Health Research Policy and Systems		2018
Multi-intervention comparisons	Piggott et al. [38]	Journal of Clinical Epidemiology		2021
GRASP	Khalifa et al. [39]	BMC Medical Informatics and Decision Making	Predictive tools for clinical decision support	2019
ISAT	Milat et al. [40]	Health Research Policy and Systems	Decision support tool for health policy makers and implementers	2020
PRECIS	Thorpe et al. [41]	Canadian Medical Association Journal	Pragmatic vs. exploratory trials for trial designers	2009
PR-Tool	Koppenaal et al. [42]	Journal of Clinical Epidemiology	Applicability of individual and SR of trials	2011
PROBAST	Moons et al. [43]	Annals of Internal Medicine	Risk of bias and applicability of prediction model studies	2019
RE-AIM	Glasgow et al. [22, 44]	American Journal of Public Health Health Education Research	Evaluate and report on internal and external validity, and impact of health promotion programs	1999 2006
RoB 2	Sterne et al. [7]	British Medical Journal	Risk of bias in randomized trials	2019
RoBINS-I	Sterne et al. [6]	British Medical Journal	Risk of bias in non-randomised studies of interventions	2016
STP	Lavis et al. [45]	Health Research Policy and Systems	Applicability of the findings of a systematic review	2009
WHO-INTEGRATE EtD	Stratil et al. [46]	Cost Effectiveness and Resource Allocation	Decision criteria for health decision making	2020
Almeida et al. [47]		Int J of Tech Assessm in Health Care	Translation of HTA evidence into policy	2019
Atkins et al. [48]		Journal of Clinical Epidemiology	Applicability when comparing medical interventions for SR	2011
Berger et al. [49]		Value in Health	Relevance and credibility of observational studies for health care decision making	2014
Bonell et al. [50]		British Medical Journal	Generalizability in trials of health interventions	2006
Bornhoft et al. [51]		BMC Medical Research Methodology	Evaluation of clinical studies on external and model validity	2006
Burford et al. [52]		Journal of Clinical Epidemiology	Applicability of findings in systematic reviews of complex interventions for SR	2013
Green et al. [17]		Evaluation and the Health Professions	Relevance, generalizability, and applicability of research	2006
Gruen et al. [53]		Bulletin of the World Health Org	Generalizability of studies in LMIC for SR	2005
Linan et al. [54]		Journal of Evidence-Based Medicine	Clinical applicability of CPG	2020
Polus et al. [55]		Int J of Tech Assessm in Health Care	Applicability of a technology in the context of HTA	2017

Notes. *CPG* clinical practice guidelines, *HTA* health technology assessment, *LMIC* low- and middle- income countries, *SR* systematic review

**Fig. 2:**
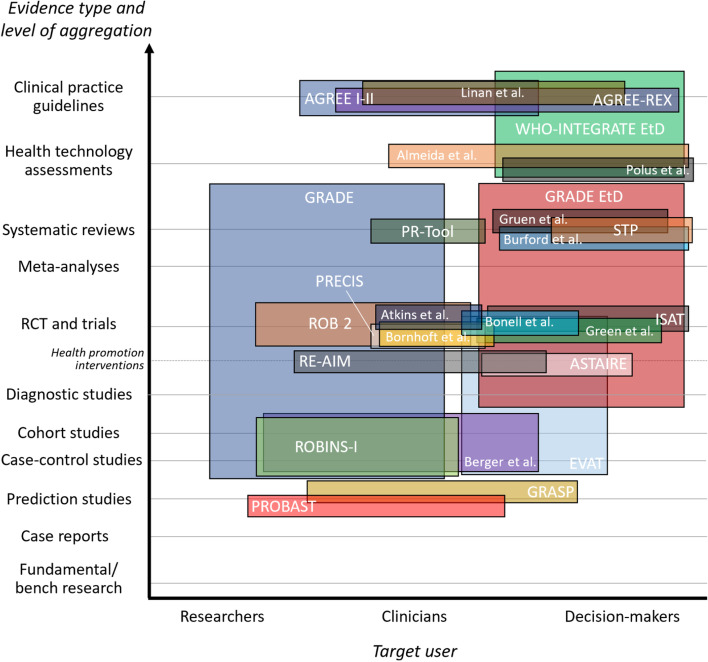
Existing frameworks for the appraisal of applicability according to evidence type and target user

**Fig. 3:**
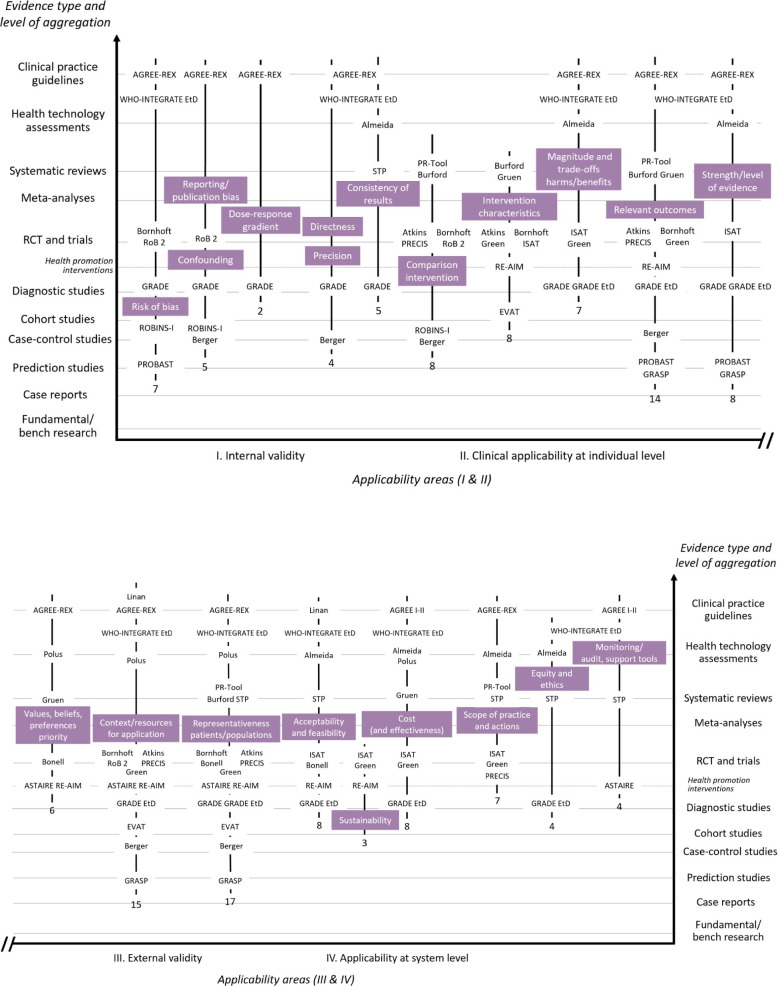
Criteria used to appraise applicability by framework, frequency, and according to evidence type and applicability domain. The number under each vertical line indicates the count of frameworks (*n*=26) featuring the criterion

Although the categories within these dimensions are not mutually exclusive, they allow the mapping and synthesis of the multiple purposes and understandings of applicability, as illustrated in Figs. 2 and 3.

#### Mapping of frameworks and synthesis of criteria

Figure 2 maps the 26 frameworks according to the evidence type appraised and the primary intended target user. For most frameworks, the scope of the evidence appraised was directed at a single level of aggregation (e.g. prediction studies [39, 43], trials [7, 17, 41, 48, 50, 51], CPG [29, 31, 54, 57]); a few frameworks bridged evidence types such as the GRADE [11] framework which examines findings from case-control and cohort studies to systematic reviews. Most frameworks were intended for multiple stakeholders (researchers, clinicians, decision-makers), but none encompassed all three. There was a qualitative association between the level of aggregation of evidence and the primary intended users: as the frameworks appraised increasingly aggregated evidence (e.g., HTA or CPG) the target users tended towards decision-makers, whereas frameworks pertaining to prediction and observational studies were more focused on researchers, with in the middle, frameworks on trials focused mostly on clinicians.

Fig. 3 summarizes the criteria extracted from the frameworks. Across all frameworks, 21 criteria were synthesized and qualitatively mapped to evidence type appraised and the applicability areas. Although there was overlap of areas of applicability, 7 criteria fell under internal validity (i.e., risk of bias, confounding, reporting bias, dose-response gradient, precision, directness, consistency of results, and comparison intervention). Clinical applicability at the individual level directly encompassed 5 criteria (i.e., comparison intervention, intervention characteristics, magnitude and trade-offs of harms and benefits, relevance of outcomes, strength/level of evidence); and external validity considered 3 critical criteria (values, beliefs, preferences priority; context and resources for application; representativeness of patients and populations). The latter two criteria along with relevant outcomes were the most frequently featured criteria across frameworks. Finally, six criteria related to applicability at the system level (i.e., acceptability and feasibility, sustainability, cost and cost-effectiveness, scope of practice and actions, equity and ethics, monitoring/audit and support tools). There was a qualitative association between criteria in frameworks about higher level of aggregation of evidence and applicability at the system level. Existing frameworks on clinical applicability span multiple target users, evidence types, and areas of applicability. Applicability holds different meanings whether one is a researcher, clinician, or decision-maker, and is ascertained using different set of criteria depending on the type of evidence and whether internal validity, clinical applicability, external validity, or system applicability is emphasized. Our proposed framework focuses on the clinical perspective and aims to assist clinicians when evaluating all types of primary study results (from fundamental research to RCT and trials) to determine whether and how these apply to clinical practice.

### Proposed framework: the framework to appraise the clinical applicability of studies (FrACAS) and VICORT checklist

#### Operational definition and classification of “clinical applicability:” the FrACAS framework

**Fig. 4:**
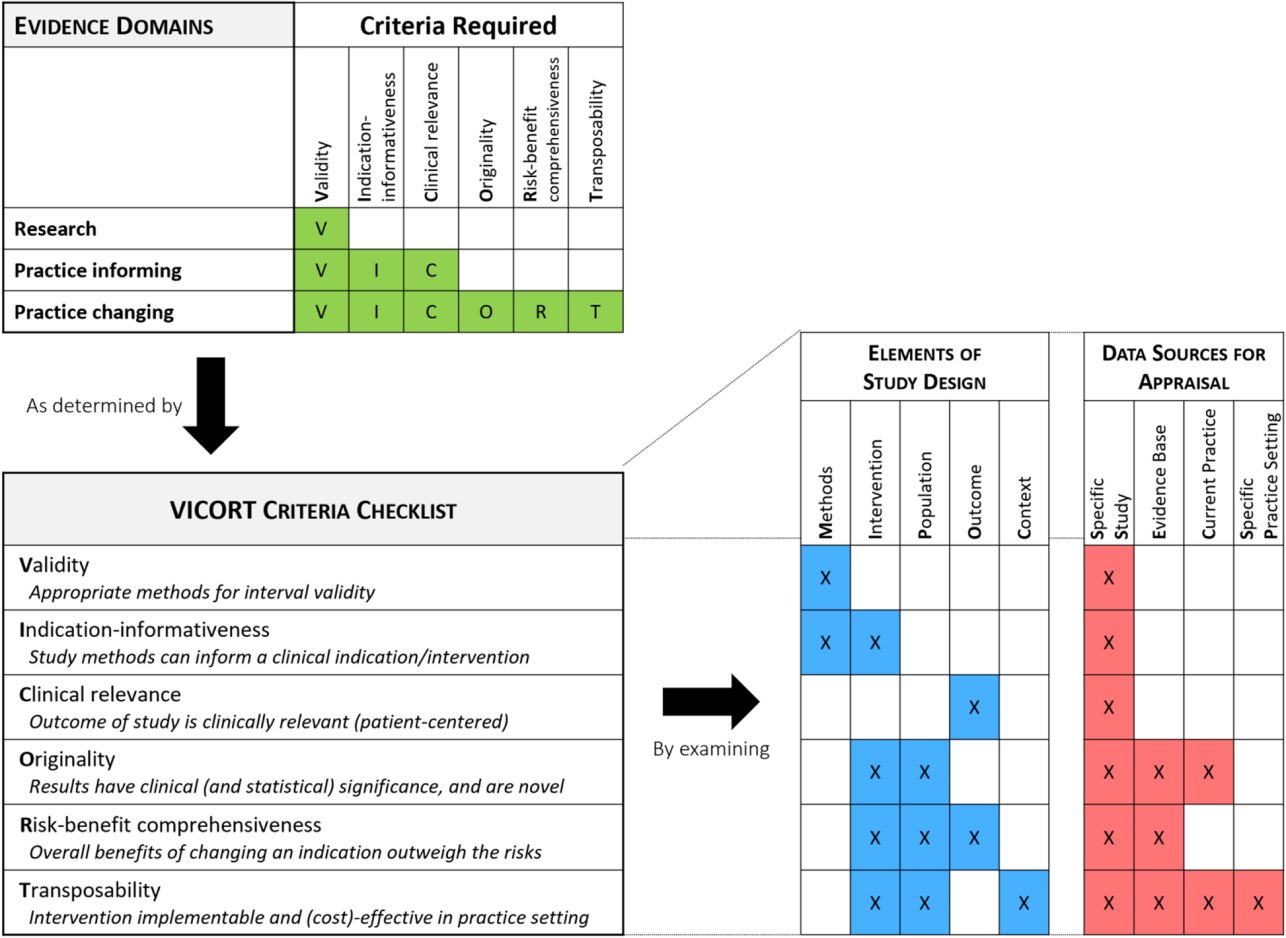
Framework for appraising clinical applicability of studies (FrACAS) and VICORT criteria

FrACAS uses an operational definition of clinical applicability that classifies a study according to the following questions: “are these research results valid?”, “can these results inform [my] practice?”, or “do these results change [my] current practice?”. As shown in Fig. 4, studies are classified in one of three evidence domains: research, practice-informing, or practice-changing domains, based on six criteria that examine study design elements and related data sources.

#### Criteria for appraisal and classification in FrACAS: the VICORT checklist

The six criteria that determine study classification in FrACAS are: Validity, Indication-informativeness, Clinical relevance, Originality, Risk-benefit comprehensiveness, and Transposability (VICORT checklist). Study findings are considered progressively more informative and practice changing as they sequentially meet these criteria. Table 2 presents each criterion’s definition and comparisons with criteria synthesized in the review.

**Table 2:** VICORT criteria definition and relation to other epidemiological concepts

VICORT Criteria	Definition	Related criteria from literature synthesis	Relation to other epidemiological frameworks or concepts
**V**alidity	Methods are appropriate for internal validity of results: ∙ Experimental evidence generated is not subject to randomization, blinding, protocol deviation, missing data, or measurement issues. ∙ Observational evidence generated is not subject to confounding, information, and endogenous selection biases.	Confounding Consistency of results Dose-response gradient Precision Reporting bias Risk of bias Strength and level of evidence	Quality assessment tools, e.g., - Cochrane risk-of-bias tool [7] - Risk of bias in non-randomised Studies of Interventions) [6] - Newcastle-Ottawa Scale [58]
**I**ndication-informativeness	Study methods provide clinicians with evidence to determine a clinical indication in specific individuals. Informativeness for a clinical indication requires a well-defined intervention whose effect can be identified from the study results, i.e.: 1. A trial of an intervention (experimental study) in specific/eligible individuals; **OR** 2. An observational study of an exposure where: A) A well-defined intervention for specific individuals (those with the exposure) exists, **AND** B) That the effect of this well-defined intervention be correctly identified (independent effect of the intervention); **OR** 3. An observational study where there is an intervention on specific individuals and where absolute results for outcomes are explicitly reported. (informativeness for the outcome of an intervention criterion – allows contrast between intervention in specific individuals and envisioned natural history under no intervention in those individuals).	None	Counterfactuals [59, 60] Well-defined intervention, consistency assumption of causal inference [26, 61]
**C**linical relevance	Primary outcome of the study is clinically relevant, i.e., the outcome is at a minimum clinically informative, and ideally, patient centered.	Directness Relevance of outcomes	Surrogate outcomes [62] Overdiagnosis [63] Patient-centered outcomes research [13]
**O**riginality	**Significance**. Study results achieve clinical (not only statistical) significance (e.g., a relevant magnitude of effect); **AND** **Novelty**. Study results are novel when compared to current evidence base and practice.	Comparison intervention Intervention characteristics Magnitude (effect size) and trade-offs of harms and benefits	Clinical vs sole statistical significance Dichotomization vs magnitude of effect and confidence intervals [64, 65]
**R**isk-benefit comprehensiveness	Overall benefits of changing an indication (either the intervention or the population of individuals in which the intervention is indicated) comprehensively outweigh the risks.	Magnitude and trade-offs of harms and benefits	Net benefit - Generic health state measures Relative vs absolute measures [66, 67]
**T**ransposability	The clinical indication/intervention is implementable and (cost-) effective in the specific practice setting.	Acceptability and feasibility Context and resources for application Cost and cost-effectiveness Equity and ethics Monitoring/audit and support tools Representativeness of patients and populations Scope of practice and actions Sustainability Values, beliefs, preferences priority	Generalizability and transportability [14, 22, 64] Cost-effectiveness analysis [21]

#### Validity

Validity is the criterion most discussed, established, and assessed by researchers and clinicians [2, 3]. Internal validity is a necessary criterion for study findings to be considered research evidence. As our review shows, most quality assessment tools, including the Cochrane Risk-of-Bias tool (RoB 2) [7] and the Risk Of Bias In Non-randomised Studies of Intervention (ROBINS-I) [6], focus on the validity of methods (randomization, blinding, and missing data; confounding, information, and endogenous selection bias). The importance of validity in general applicability of study results is highlighted by the 7 validity-related criteria shown in Table 2. When considered outside of the traditional epidemiology and medical research contexts, the scope of validity may vary by scientific disciplines. As a general term, validity may encompass other criteria such as clinical relevance and elements related to transposability (e.g. in psychology and medical education when referring to test validity and psychometrics; see below) [68–70]. Although internal validity is a prerequisite, it is not sufficient for clinical applicability.

#### Indication-informativeness

Validity ensures that estimates are unbiased. Indication-informativeness ensures that these estimates are applicable in clinical practice. Study findings produce estimates, but not all estimates can lead to action in clinical practice. To do so, the study should produce results that inform a clinical indication, i.e., an intervention in a specific population. An indication entails the identification of what clinicians should do and which population would benefit from this being done. To inform a clinical indication, a study must include a well-defined intervention whose effect is identifiable in the results (i.e., identifiability). The ability to identify and to promise the future effects of an intervention under consideration is the key criterion to achieve indication-informativeness and move from the research domain to the clinical practice domain.

Only some study designs fulfill this criterion. Firstly, randomized control trials (RCT) where an intervention is evaluated in an eligible/target population. Secondly, observational studies of an exposure for which there exists an intervention (or where one is envisioned) to remove or modify the exposure of interest [71]. If validity is ensured, the effect of the intervention can be identified and generally assumed to approximate the effect of the exposure (e.g., smoking cessation and smoking). The existence (or lack thereof) of an exposure-removing intervention is the core of the indication-informativeness criterion. HIV, smoking, atherosclerosis, frailty, and age are exposures with decreasing levels indication-informativeness since eliminating each is increasingly challenging. Third, observational studies can also inform a clinical indication by descriptively reporting absolute outcomes of an already/otherwise-indicated intervention in a specific population of interest. For example, reporting the absolute mortality following heart surgery indicated for coronary artery disease, in patients with frailty, informs this indication by allowing the counterfactual contrast between undergoing an intervention and the natural history when forgoing the intervention, in those with frailty. Of note in this scenario, the well-defined intervention is not indicated on the basis of frailty. Following these three study designs, exposures can form the basis of an indication (i.e., inform an intervention or specific population) only when they are used in a study as a selection criterion, predictor, mediator, or effect modifier, not when used as a confounder or outcome.

Indication-informativeness does not currently feature explicitly in any identified frameworks. However, it is strongly related to the widely debated requirement of well-defined interventions in epidemiology [23, 72–74]. Our framework contextualizes the presence of the well-defined intervention/consistency assumption [26, 61] as a requirement for evidence that is clinically informative and applicable, not for epidemiological evidence itself [75].

#### Clinical relevance

Epidemiological research spans a broad range of outcome types including basic science mechanisms, intermediate outcomes, and patient-centered outcomes [13]. Clinical relevance requires that study outcomes be directly relevant and informative to practice. The precise delimitation of what outcomes are informative to practice varies [13]. It may be easy to restrict measures of heart stem cell transplantation survival to being clinically non-informative, but cholesterol levels, coronary calcium scores, atherosclerotic cardiovascular disease hospitalization, mortality, and health-related quality of life (HRQoL) all have some clinically relevant information. Achieving full clinical relevance benefits from incorporating patient-centered outcomes, of which mortality and HRQoL are examples. Ignoring outcomes that are patient-centered has led to increased numbers of studies using surrogate outcomes with unclear patient benefit and potential overdiagnoses [27, 62]. Clinical relevance in FrACAS is related to the directness [11, 14] and relevance of outcomes criteria identified in our review.

#### Originality: clinical significance and novelty

The originality criterion comprises significance and novelty. Under our framework, significance centers on demonstrating a clinically meaningful magnitude of effect (effect size), not only statistical significance [64]. Even if results are clinically meaningful, they can only alter current practice if they are novel compared to the current evidence base and standard practice, as shown in Fig. 4. Appraising novelty requires contrasting study results with a careful examination of the cumulative substantive evidence (e.g., reviews, practice guidelines) and current practices. Appraisal is thus practice-setting dependent. Under an evidence-based research approach, the broader context of study question and results should be systematically considered in the planning and interpretation of the study itself [12, 76]. The novelty of a study involves changing an intervention-population coupling: this requires altering (i.e., adding or removing) an intervention in a specific population or, conversely, modifying a specific population as eligible for an intervention. For example, finding that exercise benefits older adults with frailty may not be novel since exercise is already recommended to older adults in general. The difference between statistical and clinical significance (magnitude of benefits) has been highlighted in frameworks [11, 17, 18, 31, 40, 46, 47], but the importance of the novelty of findings to alter practice has not. The lack of novelty may explain why some prediction studies do not alter practice: if all modifiable predictive exposures are already addressed in standard care, then no new indication can be identified.

#### Risk-benefit comprehensiveness

Will altering an indication in current practice prove comprehensively beneficial to patients? Two sides must be examined: first, the intervention and displaced alternatives and, secondly, their summary net effect on overall outcomes [77]. Comparing a drug to placebo will not displace the same alternatives as comparing a drug with another active agent; if the study outcome is condition-specific at the expense of remaining patient-centered, important complications or outcomes may be overlooked that would outweigh the observed benefit. The withdrawal of the nonsteroidal anti-inflammatory drug rofecoxib due to unanticipated cardiovascular events is one example of the importance of comprehensively considering risks and benefits [78]. The risk-benefit comprehensiveness criteria emphasizes the necessity of examining explicitly and comprehensively the magnitude and trade-offs of harms and benefits criterion identified in available frameworks [11, 17, 18, 31, 40, 46, 47]. The correct calculation of comprehensive health outcomes to estimate net-benefit requires that outcomes be integrated on the absolute scale rather than on the relative scale [66].

#### Transposability

Appraising transposability involves taking all elements of study design, including the broader context of the study, and applying them to a specific practice setting. Epidemiologists and clinicians readily consider the external validity rubrics of generalizability and transportability [25, 79, 80]. Our transposability criterion has a wider scope. In addition to considering the population and effect modifiers (effectiveness) [25], transposability includes all other facets of implementing the intervention in a given practice setting, e.g., acceptability and feasibility, cost-effectiveness, ethics, and sustainability [18, 22, 46, 48, 53]. These will vary by practice context: resource settings, income levels, healthcare systems and payers, preferences priority, etc. [18, 21, 46, 52, 81]. As these additional questions enter into the realm of implementation science and economic evaluation, they may be beyond the direct purview of epidemiological research and are not exhaustively detailed in FrACAS.

## Discussion

We identified 26 unique frameworks that appraise applicability of studies varying according to the evidence type assessed and the intended target user. Within these frameworks we synthesized 21 criteria focused on four facets of applicability (internal validity, clinical applicability at the individual level, external validity, and applicability at the population or system level). Our mapping of frameworks can help researchers, clinicians, and decision-makers select the most suitable framework depending on the appraisal question and context; selected framework may be further customized by including other synthesized criteria.

We propose a framework aiming to assist clinicians in the appraisal of clinical applicability. FrACAS shares many criteria with existing more structured and widely adopted frameworks. We believe that FrACAS is complementary to established frameworks. First, our framework creates three practical and operational domains of clinical applicability that are meaningful from a clinical practice standpoint: research evidence (i.e., does not inform clinical practice directly), practice informing, and practice changing. Rather than having the full body of existing evidence on a topic as the primary area of focus, FrACAS takes each individual study and characterizes its clinical applicability and impact, which is typically how new findings are examined and consumed in daily practice.

Next, to distinguish between level of evidence domains, FrACAS proposes two additional criteria not explicitly featured in other frameworks: indication-informativeness and originality. Many frameworks emphasize study design to determine clinical applicability and give more weight to RCT and meta-analyses than to cohort and case-control designs. The indication-informativeness criterion makes clear that it is not the study design per se that allows a study to inform and alter practice but its ability to validly inform an indication. Many health-improving interventions did not originate from experimental evidence (e.g., smoking cessation). RCT evidence has an easier claim to validity, indication-informativeness, and thus clinical applicability. However, one cannot invalidate causal inference from observational studies, only require more caution [71]. The criterion of originality is important to differentiate studies between being practice-informing or practice-changing. Determining originality (novelty and significance) is clinically consequential: practice-informing studies can go unnoticed by clinicians without major detriment since they do not alter any indication, but practice-changing studies cannot. The novelty of study results is often the prime answer to the “so what?” question of clinical applicability, following the “is it credible?” question of internal validity.

Our framework and criteria span multiple evidence types and target users, from fundamental research up to trials and, though focused on clinicians, can be relevant to researchers and decision-makers. FrACAS proposes six relatively orthogonal criteria and does not reduce them to one or two dimensions to summarize the strength or certainty of evidence [82]. FrACAS can be used as a checklist to diagnose which study design elements should be addressed for a study to change practice. Clinical translation can and does occur in the absence of one or many criteria, but we believe that careful analysis would reveal that missing criteria are assumed. We believe that the conciseness of our framework and checklist will help clinicians and trainees appraise and discuss study findings in daily practice.

Finally, our framework emphasizes the highly contextual and potentially subjective nature of appraising clinical applicability. By explicitly describing study design elements and data sources to be examined for each criterion, we show that determining practice-changing status requires the consideration of an increasing number of features. Whereas classifying articles as practice informing can be based on the appraisal of the individual study in question, a practice changing classification requires consideration of the cumulative evidence base, current standard and specific practice setting. Changing practice is an interdisciplinary and concerted effort requiring both methodological and substantive expertise.

### Limitations

Although we carried out a robust literature search, extraction, and synthesis process, we did not conduct a formal systematic review. Even if we used a very wide search strategy, we may have omitted applicability frameworks. Our review serves primarily as a map to compare frameworks and criteria rather than to examine their relative strengths and weaknesses [15, 16, 83–85]. The process of developing a conceptual framework entails some subjectivity and variability; although a formal Delphi method was not employed, we included a wide range of inputs to iterate versions of our framework (current frameworks, debates in epidemiology, multiple stakeholders, and pilot testing). This representativity and the relative overlap with existing frameworks provide face and content validity. Ultimately, the most proper test of validity and usefulness of our framework will be determined in its usage and application in the real world; further refinements may benefit from wider inclusion of patient and institutional stakeholders.

## Conclusion

Frameworks appraising applicability can be classified according to the types of evidence assessed, target users, and areas of applicability (internal validity, clinical applicability, external validity, applicability at population/system level). We proposed a concise framework focusing on clinical applicability which uses six criteria to classify studies into three evidence domains: research, practice informing, or practice changing. Our framework can be used as a tool for the design, appraisal, and interpretation of epidemiological and clinical studies to improve their clinical applicability.

## Supplementary Information

**Additional file 1: **Methods. **Figure A.1.** Flowchart for selection of articles.

## Data Availability

The datasets used and/or analysed during the current study are available from the corresponding author on reasonable request.
