# Supplementary material for: Appraising clinical applicability of studies: mapping and synthesis of current frameworks, and proposal of the FrACAS framework and VICORT checklist
Source: BMC Med Res Methodol. 2021 Nov 14;21:248. doi: 10.1186/s12874-021-01445-0 (PMC8590785; doi:10.1186/s12874-021-01445-0)
Supplement: Supplementary file 1 — Additional file 1: Methods. Figure A.1. Flowchart for selection of articles. [file 12874_2021_1445_MOESM1_ESM.docx]

# Appendix

Appendix for:

Appraising clinical applicability of studies: mapping and synthesis of current frameworks, and proposal of the FrACAS framework and VICORT Checklist

# Table of contents

[Appendix Methods. 2](#_Toc84342976)

[Figure A.1. Flowchart for selection of articles 3](#_Toc84342977)

## Appendix Methods.

| **Search query for Embase using OVID**  (("clinical practice" or decision or applica* or "clinical impact" or transferability or translat* or "external validity" or generalisability) and  (apprais* or asses* or grad* or analy* or "systematic reviews" or evaluat* or factor*) or (framework or recommendation or consensus or guide or checklist or tool* or instrument or program or questions)).ti. and  english.lg. |
| --- |

| **Search query for PubMed**  ("clinical practice" or "evidence" or "decision" or "applica*" or "clinical impact" or “transferability” or “translation” or “implementation” or “external validity”) and  ("apprais*" or "assess*" or "grad*" or “analyse” or “analyze” or “systematic reviews”)  ("framework" or "recommendation" or "consensus" or "guide" or "checklist" or "tool*" or "instrument*" or “program”) and  english[Language] |
| --- |

## Figure A.1. Flowchart for selection of articles

**
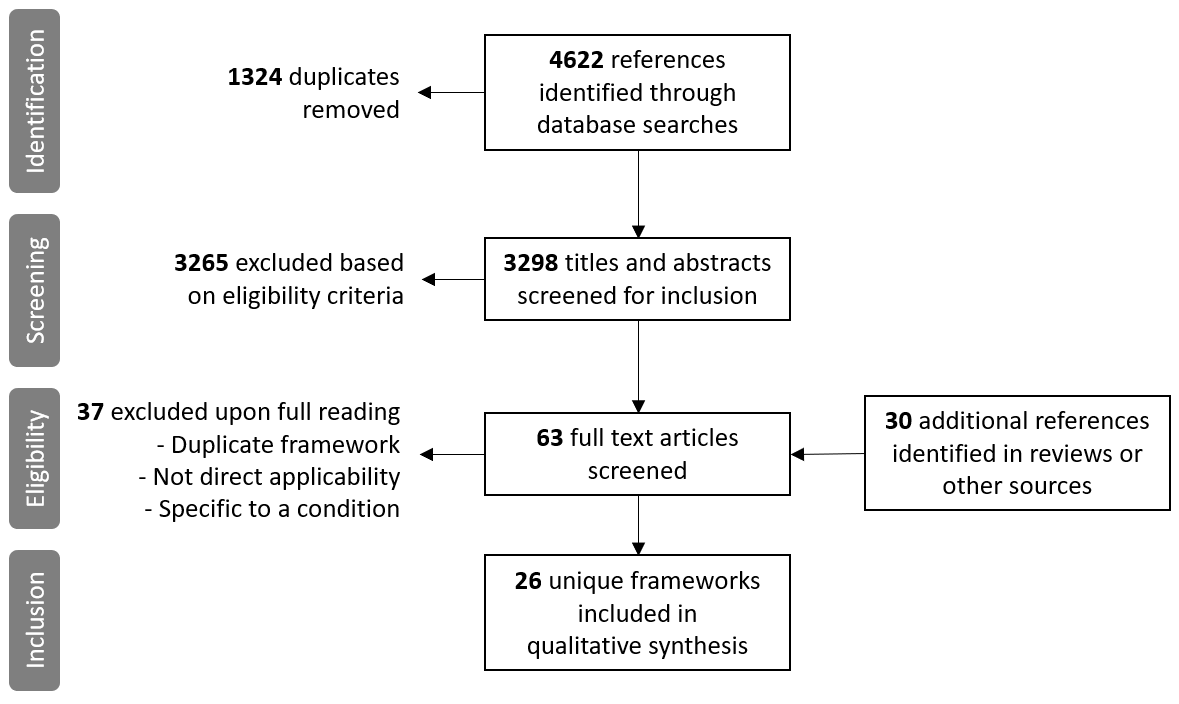
**
